# Cardiac Autonomic Control Mechanisms in the Pathogenesis of Chagas' Heart Disease

**DOI:** 10.1155/2012/980739

**Published:** 2012-10-02

**Authors:** Diego F. Dávila, Jose H. Donis, Gabriela Arata de Bellabarba, Vanesa Villarroel, Francisco Sanchez, Lisbeth Berrueta, Siham Salmen, Barbara Das Neves

**Affiliations:** Instituto de Investigaciones Cardiovasculares, Departamento de Fisiopatología, Instituto de Inmunologìa Clinica, Facultad de Medicina, Hospital Universitario de Los Andes, Universidad de Los Andes, Mérida 5101, Venezuela

## Abstract

Primary abnormalities of the autonomic nervous system had been postulated as the pathogenic mechanisms of myocardial damage, in patients with Chagas disease. However, recent investigations indicate that these abnormalities are secondary and amenable to treatment with beta-adrenergic blockers. Moreover, muscarinic cardiac autoantibodies appear to enhance parasympathetic activity on the sinus node. Therefore, the purpose of this paper is to analyze how knowledge on Chagas' disease evolved from being initially considered as a primary cardioneuromyopathy to the current status of a congestive cardiomyopathy of parasitic origin.

## 1. Introduction

 The natural history of Chagas disease is characterized by an acute phase, followed by an indeterminate or transitional stage and a terminal arrhythmic-congestive phase. This disease appears to evolve from localized myocardial damage to a clinical form of congestive cardiomyopathy, with diffuse myocardial damage [1–4]. Several hypotheses have been postulated in order to explain the mechanisms responsible for the progression of myocardial damage. The proposed mechanisms are (1) microvascular disturbances, (2) immune-mediated myocardial injury, (3) parasite-dependent myocardial aggression, and (4) primary abnormalities of the parasympathetic and sympathetic divisions of the autonomic nervous system. Microcirculatory disturbances and immune-mediated myocardial injury are prominent peculiarities of Chagas cardiomyopathy. However, the roles of these two proposed mechanisms of myocardial damage are very likely ancillary rather than fundamental to the pathogenesis of disease progression. Concerning parasite-dependent myocardial damage, due to the diversity of *Trypanosoma cruzi *populations isolated from patients presenting the same clinical form of the disease an association between the parasite's genotype and the clinical manifestations of the disease is still not definitively established. Moreover, the available data are considered insufficient to justify trypanocidal therapy as a therapeutical alternative aimed at modifing clinical outcomes. Nonetheless, the BENEFIT trial (Benefit Evaluation for Interrupting Trypanosomiasis), currently in progress, will undoubtedly provide definitive answers for this crucial therapeutic dilemma [1–4].

 The parasympathetic abnormalities were initially attributed to a direct action of the parasite on the postganglionic cardiac parasympathetic neurons. This hypothesis was postulated by Koberle in the 1950s [5, 6]. A cardiac autoimmune response, aimed at the sympathetic postganglionic fibers and to the cardiac muscarinic receptors, was proposed in the 1990s by Iosa et al. and Goin et al., respectively [7–9]. The autoantibodies, with adrenergic and cholinergic activities, would be responsible for an early sympathetic and parasympathetic dysautonomias. These two primary abnormalities of the autonomic nervous system would precede and contribute to the progression of myocardial damage and cardiac dysfunction. Recent investigations have shown, on the contrary, that these autonomic abnormalities are indeed secondary and characterized by an impairment of cardiac parasympathetic control and activation of the sympathetic nervous system [2, 10]. Moreover, muscarinic cardiac autoantibodies appear to enhance parasympathetic control of heart rate and therapeutic strategies, which antagonize the cardiotoxic effects of catecholamines, increase quality of life and survival of these patients [11, 12]. Therefore, the main purpose of this paper is to analyze how knowledge on Chagas disease evolved from being initially considered as a primary cardioneuromyopathy to the current status of a congestive cardiomyopathy of parasitic origin.

## 2. Chagas Heart Disease as a Primary ****Cardioneuromyopathy 

### 2.1. The Neurogenic Hypothesis on the Pathogenesis of Chagas Heart Disease

**Figure 1:**
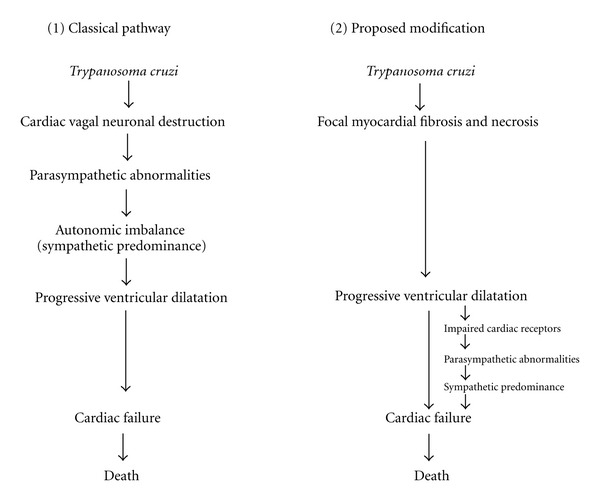
The neurogenic hypothesis on the pathogenesis of myocardial damage in chagas disease. According to the classical pathway, sympathetic activation, due to an early and selective destruction of the cardiac postganglionic neurons, would precede and induce cardiac ventricular dilatation. The proposed modification states that myocardial damage of certain extent and sequelae of the acute phase of the disease leads to progressive ventricular dilatation with impairment of cardiac receptors, parasympathetic abnormalities, and sympathetic activation (see [15]; 5 : 327-29, adapted with permission from Elsevier).

 According to the neurogenic hypothesis, the parasite would irreversibly and selectively destroy the postganglionic cardiac parasympathetic neurons during the acute phase of the disease. Sympathetic predominance would follow, exposing the myocardium to the toxic effects of catecholamines. However, Koberle's hypothesis was based on autopsies of patients who had died in advanced stages of the disease (i.e., congestive heart failure). Cardiac neuronal counts in patients, with no macroscopic evidence of myocardial damage, were either normal or minimally decreased [5, 6, 13]. Moreover, functional tests of autonomic function were normal in asymptomatic patients with no electrocardiographic evidence of heart disease and could also be normal or impaired, in those patients with abnormal electrocardiograms [14]. The results of these functional and morphological studies clearly indicated that the abnormalities of the parasympathetic nervous system were not homogenous. According to the neurogenic hypothesis most if not all Chagasic patients should have functional evidence of an impaired parasympathetic control of heart rate and abnormal neuronal counts, in the early stages of the chronic phase of the disease (i.e., intederminate stage). The presence and extent of myocardial damage, in most clinical studies carried out between 1960 and 1980, had been assessed by means of surface electrocardiography and chest X-rays [14]. We, at the Institute of Cardiovascular Research of the University of the Andes in Mérida, Venezuela, were puzzled by the heterogeneity of the results of the functional and morphological studies on the autonomic nervous system of Chagasic patients and postulated an alternative explanation for the autonomic abnormalities (Figure 1) [15]. To test this alternative hypothesis, we began a series of experimental [16–21] and clinical studies [22–24], on the functional status of the autonomic nervous system, in acutely infected laboratory animals and in Chagasic patients who were in the different stages of the natural history of the disease (acute, indeterminate, and congestive stages). The presence and extent of the myocardial damage were assessed by autopsy in the former and by left ventricular cine angiography in the latter. The results of these investigations were congruent in pointing out the following.

**Figure 2:**
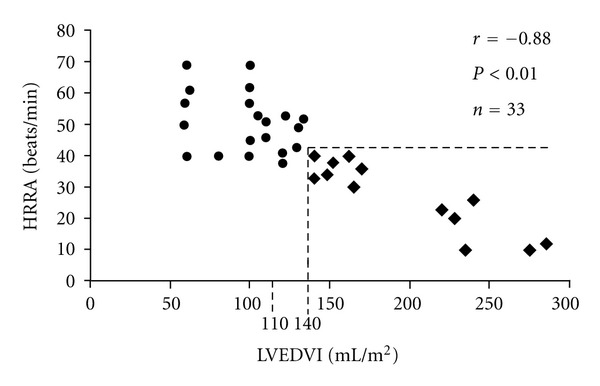
Parasympathetic abnormalities and cardiac remodelling in chagas disease; the heart rate response to atropine (HRRA) is indirectly related to left ventricular end-diastolic volume (LVEDI). Thus, parasympathetic impairment is secondary to cardiac remodelling and dysfunction (● = patients with segmental myocardial dysfunction, normal HRRA, and no ventricular dilatation; ◆ = patients with abnormal HRRA and progressive ventricular dilatation). (see [22], adapted with permission form Elsevier).

 (1) Acutely infected laboratory animals, with unequivocal evidence of chagasic myocarditis, had normal functional tests of the efferent and afferent components of the cardiac parasympathetic nervous system. (2) Asymptomatic Chagasic patients, with abnormal ectrocardiograms, could have either segmental of diffuse myocardial damage and variable degrees of left ventricular systolic dysfunction. (3) The heart rate response to atropine and to the Valsalva maneuver was normal in patients with localized myocardial damage, but these tests were impaired in the presence of diffuse myocardial damage. Furthermore, the heart rate response to atropine was significantly and inversely related to the degree of cardiac remodelling (i.e., left ventricular end-diastolic volume and ejection fraction) (Figure 2) [22]. All of these studies consistently questioned the primary nature of the cardiac parasympathetic abnormalities. It was now absolutely necessary to determine when, in the different stages of the natural history of Chagas disease, the activation of the sympathetic nervous system occurred [25, 26].

### 2.2. Abnormalities of the Sympathetic Nervous System

**Figure 3:**
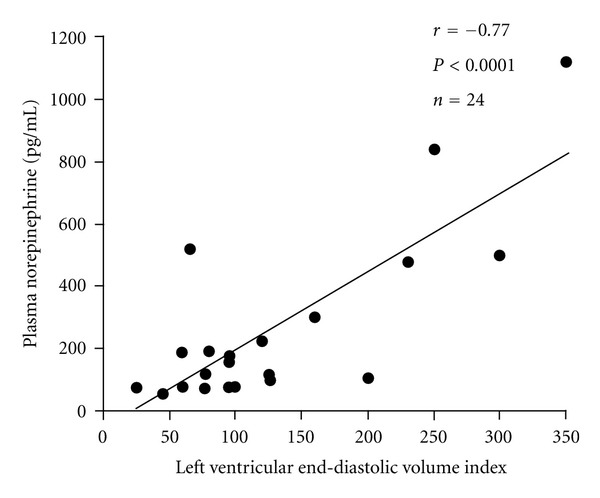
Sympathetic activation and cardiac remodelling in chagas disease. Norepinephrine serum levels are directly related to the left ventricular end-diastolic volume index. Patients with normal-sized left ventricles have no increased serum levels of norepinephrine. Thus, sympathetic activation is secondary to the process of cardiac remodelling (see [32], adapted with permission from Elsevier).

 Iosa et al. [7, 8], Goin et al. [9], and Sterin-Borda and Borda [27] considered that the sympathetic and the parasympathetic nervous systems of Chagasic patients were primarily and irreversibly damaged by the presence of cardiac autoantibodies. Chagas disease was, therefore, a primary cardioneuromyopathy. This hypothesis was based, in part, on the norepinephrine serum levels of Chagasic patients, with clinical evidence of congestive heart failure, which were found to be significantly lower than those of non-Chagasic patients [7]. At the time, we reasoned that since blood samples had been drawn from a systemic source in the former and from the coronary sinus in the latter; the methodological design of the study could be influencing the results of the investigation. Furthermore, we asked how could a patient with a primary abnormality of the sympathetic nervous system reach the stage of congestive heart failure? In the presence of low output heart failure, congestion and tissue perfusion pressure are absolutely dependent on the activation of the sympathetic and other neurohormonal systems [28]. The parasympathetic and sympathetic balance shifts towards a predominance of the latter, in non-Chagasic patients and in experimental models with asymptomatic and symptomatic left ventricular systolic dysfunction [29–31]. Consequently, Chagasic patients with impaired parasympathetic function and cardiac remodelling should also have evidence of a late and secondary activation of the sympathetic and other neurohormonal systems, as their cardiac disease progressed and congestive heart failure ensued [26]. We and other investigators found that indeed neurohormonal activation was present in the advanced stages of chagas heart disease [32, 33]. Systemic norepinephrine correlated directly and significantly with the degree of cardiac dilatation (Figure 3) [32] and coronary sinus norepinephrine was similar to that of patients with non-chagasic heart failure, due to left ventricular systolic dysfunction [33]. It was now clear to us that the abnormalities of the parasympathetic and sympathetic divisions of the autonomic nervous system were late and secondary events, in the natural history of chronic chagas disease. Despite the infectious nature of Chagas disease [6, 34, 35], the overwhelming clinical and neurohormonal similarities between chagasic and non-chagasic cardiac patients lead us to an utmost pertinent and relevant question ((Tables 1 and 2) [36–38]): should we use beta-adrenergic blockers in Chagasic patients with congestive heart failure secondary to left ventricular systolic dysfunction?

 When considering the risk factors for cardiac morbidity and mortality, prospective studies [39] provided direct and indirect evidences of neurohormonal activation in symptomatic Chagasic patients. These prospective studies indicated that a heart rate above 90 beats/min was predictive of a poor prognosis. Based on these studies and the above information already described, we decided to test the hypotheses that Chagasic patients, with congestive heart failure, would tolerate and benefit from beta-adrenergic blockers [26]. The short-term effects of the selective beta-blocker metoprolol were studied in Chagasic patients with severe congestive heart failure [40]. Patients were receiving conventional treatment and had sinus tachycardia, low systolic blood pressure, and echocardiographic evidence of severely depressed left ventricular systolic function. Mild sympathetic activation was present and most of them were in functional class IV. At the end of the fifth week of treatment with metoprolol (25 mg), the heart rate and blood pressure showed favorable and significant changes. The left ventricular ejection fraction, however, increased significantly only at the end of the tenth week of treatment with metroprol (50 mg). Similar clinical benefits had been previously shown in the 1960s by Luquez et al. in Argentina [41] and more recently by Botoni et al. [42]. However, all of these were short-term studies which did not assess the effects of beta-adrenergic blockers and mortality and rate of hospital readmissions.

**Figure 4:**
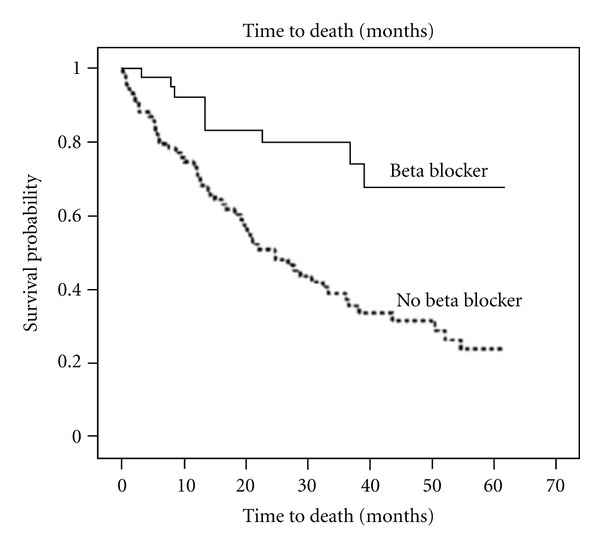
Survival probability according to use or no use of beta-adrenegic blockers. The probability of survival was significantly diminished in Chagasic patients who were not treated with beta-adrenegenic blockers. (see [43], adapted with permission from Elsevier).

 Long-term clinical investigations have been recently carried out by Brazilian investigators [43, 44]. Theodoropoulos et al., [43] followed-up patients with congestive heart failure of chagasic etiology. The design of the investigation explicitly excluded comorbidities that could contribute to the genesis of the underlying heart disease. The study was aimed to determine the predictors of all-cause mortality. To this purpose, one hundred and twenty-seven patients, who fulfilled established criteria for congestive heart failure and had received conventional treatment plus angiotensin converting enzyme inhibitors (90%) and beta-adrenergic blockers (34%) were followed up for 25 ± 19 months. multivariate cox regression survival analysis indicated that, no treatment with a beta-adrenergic blocker was a more important independent predictor of mortality than hyponatremia, left ventricular ejection fraction, and functional class. The probability of survival was significantly diminished in Chagasic patients who were not treated with beta-adrenergic blockers (Figure 4). Similar findings were more recently reported by Issa et al., [44] who compared the survival of Chagasic patients treated and untreated with beta-adrenergic blockers with that of non-Chagasic patients. These two clinical investigations are congruent in pointing out that the increased mortality of Chagasic patients, who are in the arrhythmic-congestive phase, is due to lack of treatment with neuropharmacologically active drugs. The low proportion of Chagasic patients on beta-blocker therapy may be due, in part, to the administration of targeted doses of ACEI, particularly captopril. This therapeutic strategy may lower systolic pressure and preclude the use of beta-adrenergic blockers [45]. Nonetheless, these results strongly suggest that secondary neurohormonal activation is the main underlying mechanism of disease progression, in chronic Chagasic patients [11]. Therefore, the neurogenic hypothesis [5, 6], although incorrect regarding the timing of appearance of the cardiac parasympathetic abnormalities, did envision the pathogenic role of an enhanced cardiac sympathetic drive [3, 15].

### 2.3. Parasympathetic Dysautonomia and Cardiac Muscarinic Autoantibodies

 The parasympathetic division of the autonomic nervous system is also affected by the autoimmune response to the presence of the parasite in the hearts of patients with Chagas disease [46]. A cardiac autoimmune response arises in Chagasic patients because of antigenic mimicry between the parasite and cardiac muscarinic receptors [47]. The second extracellular (o2) and the third intracellular loops (i3), of these receptors, are considered as autoimmune epitopes, in patients with chronic chagas disease [48, 49]. The autoimmune response occurs early in the natural history of the disease and it is considered to be responsible for the abnormalities of parasympathetic control of heart rate [50–54]. These investigations have demonstrated that the chronotropic responses to cardiac autonomic tests are apparently impaired in the indeterminate form of the disease. However, we [22–26] and other investigators [55] have found that Chagasic patients, who are in different stages of natural history of the disease, may have normal, abnormal [2], or even enhanced responses to conventional cardiac autonomic tests [56, 57]. Moreover, the frequency and time domain indexes of parasympathetic modulation may be depressed in the supine position, but become similar to controls in the standing position and while performing isometric exercise [58–60]. Since, muscarinic autoantibodies indirectly correlate with the high frequency component of heart rate variability [52] are highest in the presence of chronotropic insufficiency [55] and may behave as positive allosteric modulators of parasympathetic activity [61], an alternative explanation, for the “impaired” chronotropic responses to the cardiac autonomic tests, would be over stimulation with saturation of the parasympathetic system [62–65]. As stated by Benchimol-Barbosa, a continuous cholinergic effect of anti-M2 antibody, by acting on the muscarinic receptor of the sinus node cells, could slow heart rate and limit acute heart rate variations. Therefore, adjustments of heart rate in response parasympathetic efferent activity are expected to be apparently “impaired” [62]. Consequently, we have studied the cardiac chronotropic responses to the Valsalva maneuver and to dynamic exercise and correlated them with the serum levels of cardiac muscarinic autoantibodies, of Chagasic patients who were in the chronic phase of the disease [12]. Our clinical investigation included asymptomatic Chagasic patients, who were in the indeterminate and cardiac forms of the disease and had normal two-dimensional echocardiograms. Heart rate acceleration, during the early phases of the Valsalva maneuver and of dynamic exercise, was significantly diminished in the Chagasic patients. However, the heart rate changes, during the late phases of the Valsalva maneuver and of dynamic exercise, revealed a normal and enhanced response, respectively. These apparently “discordant” results would indirectly suggest the following. (1) The diminished initial heart rate acceleration during the early phases of both of these tests is indicative of reduced or impaired resting parasympathetic activity; (2) the normal or augmented heart rate recovery, at the end of these two same tests, would indicate, on the other hand, that parasympathetic reactivation is normal and even enhanced. Thus, chronotropic responses to parasympathetic withdrawal would be apparently impaired, but the chronotropic responses to parasympathetic reactivation are normal or even accentuated. How can one reconcile these apparently “discordant” results, with current knowledge on cardiac parasympathetic function in chronic chagas heart disease? 

 Antimuscarinic autoantibodies are detected eary in the natural history of chagas disease [52]. However, the serum levels of these autoantibodies do not differentiate the various forms of chagas heart disease and do not correlate with the parameters of left ventricular function. Therefore, the role of the autoantibodies in the pathogenesis of myocardial damage and disease progression is questionable [54]. Moreover, muscarinic receptors are known to be upregulated by *Trypanosoma cruzi* infection and muscarinic cardiac autoantibodies potentiate the chronotropic effects of acetylcholine on the cardiac muscarinic receptors of the sinus node [61–66]. Therefore, the postsynaptic muscarinic receptors, which mediate the negative chronotropic effects of parasympathetic activity, are numerically increased and positively influenced by *Trypanosoma cruzi *infection. In this particular context, we have found that the serum levels of the cardiac muscarinic autoantibodies correlated directly with the magnitude of cardiac deceleration, following cessation of exercise. Therefore, the more prominent heart rate recovery of the Chagasic patients could be an expression of a positive allosteric effect of the muscarinic autoantibodies on the membrane muscarinic receptor. Alternatively, these results could be due to a direct agonist effect of the autoantibodies on the muscarinic receptor and thereby potentiate early heart recovery [12]. The results of this investigation provide a plausible explanation for the heterogeneity of heart rate responses to conventional cardiac autonomic tests [55–60, 67]. A continuous cholinergic effect of anti-M2 antibody, by acting on the muscarinic receptor of the sinus node cells, may limit acute heart rate variations (i.e., heart rate responses to parasympathetic withdrawal) and simultaneously potentiate responses to parasympathetic reactivation [62–64].

 In summary, the clinical and experimental investigations discussed indicate that the abnormalities of the parasympathetic and sympathetic divisions of the autonomic nervous systems are secondary and amenable to treatment with beta-adrenergic blockers. This therapeutic strategy, although not directed at the parasite, improves quality of life and survival of patients with chagas heart disease. The cardiac muscarinic and adrenergic autoantibodies may not have a direct role in the pathogenesis of the cardiac damage [54]. Moreover, the former appears to enhance parasympathetic control of heart rate. Consequently, knowledge on Chagas disease has evolved from being initially considered as a primary cardioneuromyopathy to the current status of a congestive cardiomyopathy of parasitic origin [11, 43–45].

## Figures and Tables

**Table 1:** Clinical, radiologic, and echocardiographic characteristics of non-Chagasic and chagasic patients with advanced systolic heart failure. There was no difference in the baseline heart rate, blood pressure, and functional class (NYHA). The degree of ventricular dysfunction and dilatation was also similar in both groups of patients (see [36], with permission from Elsevier).

Characteristic	Non-Chagasic patients (*n* = 7)	Chagasic patients (*n* = 6)	*P* value
Male	6 (86%)	5 (83%)	
Female	1 (14%)	1 (17%)	
Age	54 ± 9	47 ± 10	NS
Baseline heart rate (beast/min)	116 ± 16	115 ± 16	NS
Baseline systolic pressure (mmHg)	111 ± 14	115 ± 16	NS
Baseline diastolic pressure (mmHg)	75 ± 13	83 ± 12	NS
Functional class			
III	5 (71%)	3 (50%)	
IV	2 (29%)	3 (50%)	
Cardiothoracic index	>60.0	>60.0	
Two-dimensional echocardiogram			
Left ventricular diastolic diameter (cm)	5.9 ± 0.39	6.49 ± 1	NS
Left ventricular ejection fraction	0.17 ± 0.04	0.21 ± 0.06	NS

Values are mean ± S.D., except for sex and functional class.

**Table 2:** Baseline serum levels of norepinephrine, plasma renin activity, and aldosterone in chagasic and non-chagasic patients with advanced congestive heart failure. The baseline serum levels of norepinephrine (NE), plasma renin activity (PRA), and aldosterone (ALDOST) of the chagasic patients were not different from those of the non-chagasic patients (see [36], with permission from Elsevier).

	Non-Chagasic patients (*n* = 7)			Chagasic patients (*n* = 6)		
	NE	PRA	ALDOST	NE	PRA	ALDOST
	2698	2.33	255	1570	6.30	166
	824	6.40	326	890	10.50	363
	1211	6.57	636	2800	16.87	518
	2149	2.20	107	4827	35.57	457
	1052	3.40	918	2000	1.08	147
	1291	9.70	—	1486	—	—
	975	—	—			
Mean ± S.D.	1.457 ± 695*	5.10 ± 2.96	448 ± 325**	2262 ± 1404*	14 ± 13*	330 ± 168**
Confidence interval (95%)	942; 1972	5; 10	312; 583	1705; 2818	9; 19	194; 465

NE: plasma norepinephrine (pg/mL); PRA: plasma renin activity (ng/mL per h); ALDOST: plasma aldosterone (pg/mL). *P < 0.001 as compared to normal controls. **P < 0.05 as compared to normal controls.
